# Administration of spruce bark (*Picea abies*) extracts in young lambs exhibits anticoccidial effects but reduces milk intake and body weight gain

**DOI:** 10.1186/s13028-022-00629-y

**Published:** 2022-04-23

**Authors:** Berit Marie Blomstrand, Heidi Larsen Enemark, Håvard Steinshamn, Inga Marie Aasen, Juni Rosann Engelien Johanssen, Spiridoula Athanasiadou, Stig Milan Thamsborg, Kristin Marie Sørheim

**Affiliations:** 1 Norwegian Centre for Organic Agriculture, Gunnarsveg 6, 6630 Tingvoll, Norway; 2 Department of Animal Health and Food Safety, Norwegian Veterinary Institute, Ås, Norway; 3 Division of Food Production and Society, Grassland and Livestock, Norwegian Institute of Bioeconomy Research, Gunnarsveg 6, 6630 Tingvoll, Norway; 4 Department of Industry, Biotechnology and Nanomedicine, SINTEF, Trondheim, Norway; 5 Animal and Veterinary Sciences, Scotland’s Rural College, Edinburgh, UK; 6 Department of Veterinary and Animal Sciences, University of Copenhagen, Frederiksberg, Denmark

**Keywords:** Coccidia, Coccidiocide, *Eimeria*, Industrial by-products, Sheep

## Abstract

**Background:**

*Eimeria* spp. are widespread apicomplexan parasites known to cause coccidiosis in livestock, resulting in reduced animal welfare and productivity, particularly in sheep. The treatment options are limited, and there is an emerging development of resistance against registered pharmaceuticals. Spruce bark is rich in plant secondary metabolites (PSM), such as condensed tannins, which are bioactive compounds previously shown to have antiparasitic activity. Here, we examined the anticoccidial properties of bark extract of Norway spruce (*Picea abies*) against a field isolate of ovine *Eimeria* spp. by treating *Eimeria*-infected pre-ruminant lambs with water-extracted bark daily for 12 days. We hypothesised that the bark extract would reduce the faecal oocyst excretion and, consequently, the severity of diarrhoea.

**Results:**

Oral administration of spruce bark extract significantly reduced the excretion of *Eimeria* oocysts in milk-fed lambs post treatment till the end of the trial 22 days post infection. This difference in oocyst excretion between the treated and the untreated infected animals increased with time.

Compared to the untreated and the sham-infected control group, the group treated with bark extract had softer faeces and reduced milk intake during the treatment period. After discontinuing the treatment, the treated animals got a more solid and formed faeces compared to that of the untreated control group, and the milk intake increased to the level of the sham-infected, untreated control group. The bark extract treated animals had a lower body weight and a lower mean daily body weight gain throughout the whole duration of the experiment.

**Conclusions:**

Bark extract from Norway spruce showed marked anticoccidial properties by reducing the faecal oocyst count and associated diarrhoea in young lambs. Simultaneously we experienced detrimental effects of the treatment, displayed as reduced feed intake and daily body weight gain. Therefore, we suggest conducting similar studies with lower bark extract dosage to explore the possibilities of a better trade-off to reduce the negative impact while maintaining the antiparasitic effect.

**Supplementary Information:**

The online version contains supplementary material available at 10.1186/s13028-022-00629-y.

## Background

Coccidiosis is a common disease of livestock caused by various protozoa genera belonging to the phylum *Apicomplexa*. In small ruminants, the monoxenous parasites of the genus *Eimeria* have the potential to negatively impact animal welfare and productivity by causing diarrhoea, reduced growth, and increased mortality, particularly in young animals [1]. Oocysts excreted in the faeces are infective within a few days depending on the parasite species and the temperature, and naïve animals are infected by ingesting sporulated oocysts.

*Eimeria* spp. are common causes of diarrhoea in lambs in Norway [2, 3]. Eleven ovine *Eimeria* spp. have been described, of which *E. crandallis* and *E. ovinoidalis* are considered major pathogens [4]. In Norway, coccidiosis is mainly related to spring grazing and appear 2–3 weeks after release to pasture [3, 5].

To our knowledge only two pharmaceuticals are available for treatment of coccidiosis in mammalian livestock in Europe: Toltrazuril and diclazuril, and they are mainly meant for metaphylactic use [6]. A survey conducted in 2017 showed that anticoccidials were applied in > 80% of Norwegian sheep flocks, and most lambs were treated without a laboratory diagnosis or clinical signs of coccidiosis [3]. Odden et al. [7] hypothesised that uncontrolled and exaggerated use of anticoccidials in lambs may promote the development of drug resistance as previously shown for anthelmintics, and in 2018 anticoccidial resistance against toltrazuril was detected for the first time in ovine coccidia.

Several review articles have pointed out that plant secondary metabolites (PSM) possess antiparasitic properties, both in vitro and in vivo [8–15]. For instance, condensed tannins (CT) have proven antiparasitic effects [16–21]. Few studies have tested the activity of different PSM against *Eimeria* spp. in vivo [10, 22–26], and even fewer have tested CTs against coccidia infections of sheep [27–29]. Bark extracts from coniferous trees are rich in CT, and a recent study has shown that pine extracts have in vitro activity against *Cryptosporidium parvum*, another apicomplexan parasite [16, 30]. To our knowledge, CT-containing bark extracts from Norway spruce (*Picea abies*) have not previously been tested systematically against ovine *Eimeria* spp. in vivo in milk feeding lambs this young.

Norway has a large forestry industry. A production volume of 7.21 million m^3^ spruce (*P. abies*) and pine (*Pinus sylvestris*) logs utilised in the sawmill and wood processing industry was estimated for 2019, which is equivalent to a total of 721,000 m^3^ wet bark [31]. The possibility to exploit large amounts of excess bark from the Norwegian forestry industry offers a strong incentive to further explore novel approaches to control coccidiosis. In this study, we assessed the anticoccidial effect of a water-based extract of bark from Norway spruce (*Picea abies*) in milk-fed lambs. We hypothesized that bark extract would reduce the faecal oocyst excretion and consequently reduce the severity of diarrhoea in lambs infected with mixed *Eimeria* spp.

## Methods

### Animals

To ensure parasite-free experimental animals, a total of 24 lambs of the Norwegian White Sheep breed (“Norsk kvit sau”) where removed from their mothers at birth in April 2019, immediately washed with Optima pH 4 soap (Optima Produkter AS, Norheimsund, Norway) and dried with clean towels before being placed in a parasite free, indoor housing area (Deluxe SL calf hutches, Agri-Plastics, Galway, Ireland) [32]. Mean body weight (BW) of all lambs at birth was 4.7 kg ± 0.18 (mean ± SEM).

All experimentation was conducted in line with FOTS Norwegian Food Safety Authority, license number 18555, according to The Federation of European Laboratory Animal Science Associations (FELASA) guidelines and recommendations.

### Study design

The lambs were grouped in blocks by sex and birth weight and randomly allocated into three experimental groups (n = 8) within a week after birth: IB (infected animals treated with bark extract), IC (infected, untreated control group), and SC (sham-infected, untreated control group; for milk consumption and BW comparison). There was no difference in mean birth weight between the groups (*P* > 0.05). Animals in IB and IC, housed in pairs or three together (0.7–1 m^2^ per lamb) and the lambs in SC in one group of eight (1 m^2^ per lamb), were acclimatised in their respective huts prior to infection.

The lambs were observed twice daily with regards to health status and adverse reactions to infection and bark extract treatment. In case of adverse reactions to the bark extract treatment, we would reduce the bark extract concentration and/or the treatment frequency. If considered necessary, the animals would receive the appropriate treatment, i.e. electrolyte solutions per os. If no response was obtained, the animals would be removed from the trial.

### Parasite infection

The field isolate of mixed ovine *Eimeria* spp. was obtained from 3 to 6 weeks old, naturally infected lambs housed at the Norwegian University of Life Sciences in Sandnes, Norway. The oocysts were recovered from lambs 10 months prior to this study and stored at 2–7 °C until use. The oocysts were purified according to Eckert et al. with some modifications [32]. In short, faeces were mixed with tap water (1:10) for 30 s in a blender and filtered through a sieve (250 µm pore size). The fluid was collected in 50 mL tubes and centrifuged (1550×*g*, 5 min). Then, the top 35 mL was removed with a syringe and discarded, and flotation fluid (concentrated salt-sucrose fluid, specific gravity 1.28) was added to the precipitate (3:1) and thoroughly mixed. The mixture was left for 30 min for the oocyst to float. Subsequently, the upper 15 mL containing the oocysts were collected with a syringe and washed twice in tap water (1550×*g*, 5 min). Finally, the oocysts were washed in phosphate buffered saline (PBS) (1550×*g*, 5 min), poured into a borosilicate bottle, and left to sporulate for 10 days under constant aeration at room temperature using an aquarium aeration device.

The oocyst concentration and degree of sporulation was calculated using a modified McMaster method with a sensitivity of 5 oocysts per gram, and the solution was diluted down to 20,000 sporulated oocysts/mL in tap water and stored at 2–7 °C until use the following day [33, 34]. Speciation of the inoculum was done by examining > 500 oocysts and revealed 62% highly pathogenic *Eimeria* spp.: 54% *E. ovinoidalis* and 8% *E. crandallis*. The remaining 38% consisted of *E. parva* (18%), *E. faurei* (16%), *E. pallida* (1%), *E. ahsata* (1%), *E. weybridgensis* (< 1%), *E. bakuensis* (< 1%), and *E. intricata* (< 1%).

At the age of 23–26 days (D0), IB and IC were infected for three consecutive days by oral gavage (gastric tube) with 100.000 *Eimeria* oocysts (5 mL) per day [32]. We aimed for the experimental animals to have mild to moderate clinical symptoms of coccidiosis. SC was drenched with 5 mL tap water (Table 1).

**Table 1:** Experimental timeline showing time of infection, bark extract drenching, weighing, and faecal sampling and scoring

Project day	*Eimeria* oocyst infection	Bark extract drenching	Weighing	Faecal sampling	Faecal scoring
0	IB, IC	IB	All	All	All
1	IB, IC	IB			
2	IB, IC	IB			
3		IB			
4		IB			
5		IB			
6		IB			
7		IB	IB, IC		
8		IB			
9		IB	SC	All	All
10		IB			IB, IC
11		IB			IB, IC
12				All	IB, IC
13					
14				All	IB, IC
15				IB, IC	IB, IC
16				IB, IC	IB, IC
17				IB, IC	IB, IC
18				IB, IC	IB, IC
19				IB, IC	IB, IC
20				All	All
21			All		
22			Slaughter	All	All

*IB* infected lambs treated with bark extracts, *IC* infected, untreated control animals, *SC* sham-infected, untreated control animals

### Feeding

The lambs were fed high-quality sheep colostrum stored frozen (− 80 °C) from the previous lambing season. Good quality barn dried hay (grass-clover mixture) harvested in 2018 was purchased from a local farmer, allocated in plastic bags of 10 kg and frozen at − 25 °C for eight weeks (decontamination). The fodder was stored in the bags until feeding. The milk replacer (Pluss Ulla) and concentrates (FORMEL Lam) were purchased from the feed supplier Felleskjøpet (Oslo, Norway). Each lamb was dosed with 150 mL colostrum within 1 h post-partum, followed by five meals of 150 mL within the first 24 h of their lives. From the second day after birth, the lambs were fed milk replacer at the amounts shown in Table 2, and water, hay, and concentrate ad libitum.

**Table 2:** Quantity (L/meal and L/d) of milk replacer offered to the lambs during the experiment

Week number	1	2	3	4	5–7
L/meal	0.3	0.5	0.7	0.5	0.4
L/d	0.9	1.5	2.1	1.5	1.2

### Bark extraction and determination of CT concentration

Bark from *Picea abies* (Norway spruce) was collected from a sawmill located in Møre og Romsdal, Norway (Bøfjorden sag AS, Surnadal) in March 2019. The bark was obtained by ring debarking, air dried to approximately 40% dry weight and milled to 10–30 mm particle size with an apple grinder.

The dry bark (66 kg) was divided in two batches and each batch was extracted twice for 1–1.5 h in a stirred tank at 80 °C, using 1000 L water in step 1 and 850 L in step 2, which corresponded to 28 L tap water per kg dried bark. After each extraction step, stirring was turned off to let the bark sink. The liquid phase was collected from the top by pumping and transferred to a holding tank. The combined extract (approximately 1300 L) was evaporated in a mechanical vapour recompression evaporator with forced recirculation (Epcon, Epcovap MVR 4) to a final concentrate volume of 82 L before freeze-drying.

Total CT in the extract was quantified by the butanol-HCL assay with cyanidin-HCl as standard: the freeze-dried extract and cyanidin-HCl (standard) were dissolved in methanol (80% in water) and analysed using the conventional reagent without acetone, 2.5 h, and absorbance reading at 545 nm [35]. The extract yield was 118 mg dry matter (DM)/g dry bark, and the analysed CT yield was 5.8 mg CT/g dry bark. The concentration of CT in the bark extract was 49 mg CT/g DM extract.

The dried extract was stored at − 20 °C until use.

### Bark extract drenching

Based upon previous trials, we decided to dose the lambs with a bark extract equivalent to 0.1% CT/kg metabolic bodyweight (mBW)/d [24, 28]. Initially, we mixed 17 g dry bark extract (equivalent to 0.85 g CT, ¼ of the planned daily dose) with 250 mL milk replacer and offered this to the lambs to drink voluntarily. As the animals refused to drink this mixture, we chose to administer the bark extract dissolved in tap water with a stomach tube. Immediately prior to use, the bark extract was dissolved in tap water. From D0, IB lambs were drenched daily for 12 days. Each treatment was planned to be given as ca 250 mL (depending on BW) dissolved extract at the dose shown in Table 3. D0, IB was drenched once with CT equivalent to 0.05% of mBW in one meal (~ 250 mL dissolved extract). This dose was repeated in the morning on D1. The lambs showed reduced appetite and experienced discomfort, hence on the evening of D1 we reduced the volume given by half. This clearly reduced the discomfort of the animals. Because of the high viscosity of the extract, the solution was difficult to administer, and for the fourth extract administration (D2) we decided to dilute the extract by half. To acclimatise the animals to the treatment, this was given once daily D2–D4. From D5, this amount of extract was given twice daily and consequently the final volume per day was approximately 500 mL with a CT concentration of approximately 7.6 mg CT/mL, a CT dose of 0.05% of mBW (Table 3). The dosage was adjusted with each weighing of the animal. IC and SC were given the equivalent volume of tap water.

**Table 3:** Concentration of condensed tannins (CT) in bark extracts dosed (mg/mL) and the daily dosing of CT as percentage of lamb metabolic body weight (mBW)

Experiment day	Number of daily doses	Daily dose in % CT of mBW	CT concentration, mg/mL
0	1	0.05	15.4
1	2	0.075	13.0
2–4	1	0.025	6.7
5–6	2	0.05	6.7
7–10	2	0.05	7.3
11	1	0.025	10.3

### Sampling and laboratory analysis

**Fig. 1:**
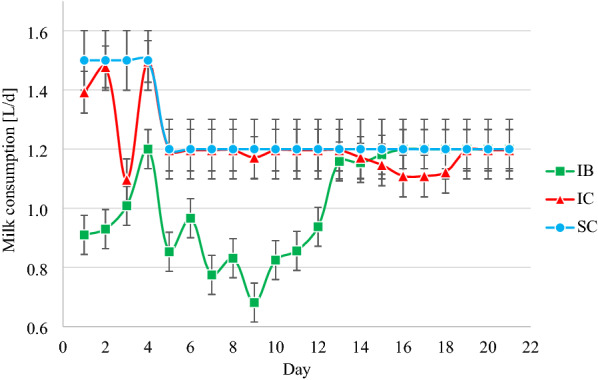
Mean daily milk consumption for all three treatment groups. *IB* Infected lambs treated with bark extract, *IC* Infected, untreated negative control group, *SC* Sham-infected, untreated control group. Error bars standard error of the means

Individual faecal samples from each lamb were collected directly from rectum on D0, D9, D12 and daily from d14 (Table 1). The faecal consistency was evaluated D0 and daily from D9 on a scale from one to five (1 = hard pellets, 2 = soft, sticky pellets, 3 = soft, paste-like with no pellet structure, 4 = watery, 5 = watery with blood and/or intestinal casts) [36]. A score > 3 was considered as diarrhoea. The faecal samples were stored in 40 mL polypropylene screw-cap containers (VWR, Avantor®) at 2–7 °C until analysis. The oocyst excretion was quantified as mentioned above using a modified McMaster method [33, 34]. All lambs were weighed at birth (D − 26 to D − 23) and on D − 6, D0, D7, and D21. Due to practical challenges, SC was weighed at D9 instead of D7. The appetite was determined by registering the daily individual milk intake (Fig. 1). The health status of the lambs was evaluated twice daily by visual inspection. We judged the animals’ posture at resting and at movement, and behaviour was evaluated on the animals’ preference for playing, eating, exploring the environment, and other physiological habits. If the general constitution of the animal was assessed to be within the physiological limits, we judged the animal to be healthy. At any deviation from expected physiological posture, behaviour, or habitus, we examined the animal more thoroughly by measuring the rectal body temperature, evaluating the pulse frequency and quality, appraise the mucus membranes, auscultating thorax and abdomen, and palpating abdomen and relevant superficial lymph nodes. All deviations were registered, and appropriate measures were taken with regards to animal health and welfare. On D22, the animals were transported to a nearby abattoir and euthanised using a captive bolt gun followed by exsanguination.

### Statistical methods

The faecal excretion of *Eimeria* oocysts in infected lambs generally follows an exponential curve before it flattens out and finally starts declining [37]. We assumed we could describe the faecal oocyst excretion per g of faeces (OPG) by the following logistic equation: $Y = Ce^{{k\left( \frac{1}{t} \right)}}$, where $C$ is the constant, e is the natural constant, $k$ is the growth rate, and $t$ is the time (day). We transformed the exponential expression into a linear equation: $Y1 = a + b*t1$, where $Y1$ is $\log \left( {Y + 1} \right)$, $a$ is $\log \left( C \right)$, $b$ is $k$ depending on treatment (IB, IC or SC), and $t1$ is $\frac{1}{t}$. Data for oocyst excretion was analysed with a repeated-measure analysis for infected groups with and without bark extract, with time ($t1$ as D0 of experiment) and treatment-time interaction as fixed effects, and pen and individual as random effects. We used the least square means procedure to estimate mean values with 95% confidence intervals (CI) at each time point.

For SC, the values of faecal consistency score (FCS) were ≤ 2 for all animals and days, and there was practically no variation, hence SC was not included in the statistical modelling of FCS.

FCS was modelled as an ordinal response variable and day was treated as a continuous variable. We ended up using a statistical model for the cumulative probabilities of the response variable, FCS:

$$ P\left( {y_{ijk} \left( d \right) \le q|B_{j\left( i \right)} ,I_{{k\left( {ij} \right)}} } \right) = \frac{{\exp \left( {\mu_{q} + \alpha_{i} + \beta \cdot d + \gamma_{i} \cdot d + B_{j\left( i \right)} + I_{{k\left( {ij} \right)}} } \right)}}{{1 + \exp \left( {\mu_{q} + \alpha_{i} + \beta \cdot d + \gamma_{i} \cdot d + B_{j\left( i \right)} + I_{{k\left( {ij} \right)}} } \right)}} , q = 1, 2, \ldots , 5 $$

where *y*_*ijk*_(*d*) is FCS for individual *k*, from housing *j* within treatment *i* on day *d*. *α*_*i*_ is the main effect of treatment *i*, *β* is the main effect of day, *γ*_*i*_ is the interaction effect between treatment *i* and day, *i* = IB, IC. *B*_*j*(*i*)_ is the random effect of house *j* within treatment *i*, and *I*_*k*(*ij*)_ is the random effect of individual *k* within house *j* and treatment *i.* The *µ*_*q*_'s are intercepts, where *µ*_1_ ≤ *µ*_2 _≤ *µ*_3 _≤ *µ*_4 _≤ *µ*_5 _(*µ*_5_ = ∞). Our null hypothesis (*H*_*0*_) was that there was no effect of treatment or of treatment by day interaction on FCS: $H_{0} :\alpha_{1} = \alpha_{2} {\text{ and }}\gamma_{1} = \gamma_{2}$. Sex had no impact on FCS and was not included in the model. The model was estimated, and the hypothesis tested using the glimmix procedure in SAS (SAS 9.4, SAS Institute Inc., Cary, NC, USA). *The null* hypotheses were tested using Bonferroni adjustment.

BW and milk consumption were analysed using the glimmix procedure in SAS (SAS 9.4, SAS Institute Inc., Cary, NC, USA). Treatment (IB, IC, SC), sex (male or female), and day in experiment (continuous) and their interactions were included as fixed effects and pen within treatment as random effect. Sex had no effect and was removed from the final model. For BW, we included BW at the start of experiment (D = 0) as a co-variate. The body weight gain for each treatment was calculated from the solutions for the fixed effects (estimated regression coefficients). Bonferroni’s test was used for pairwise comparisons of LSmeans (*P* ≤ 0.05).

## Results

### Clinical observations

One lamb in the IB group showed mild signs of colic on D1, which subdued after a few minutes. D7 and D8, two lambs (IB) refused to drink milk. Instead, they were offered an electrolyte solution, which they drank voluntarily. D9, one lamb in the SC group developed mild signs of colic, which abated after a few minutes of massaging the abdomen. On D4 of the experiment, a lamb (IB) died shortly after bark extract drenching. Necropsy results suggested an intrapulmonary rather than transoesophageal administration of the bark extract, resulting in death due to asphyxiation. This lamb was immediately replaced by a lamb from SC (lamb 10), which was infected for three consecutive days and given bark extract daily for the following 12 days. Data was tested statistically both with and without data from lamb 10, with no difference in outcome, hence we decided to include data from lamb 10 in the analyses. One lamb (IB) was diagnosed with polyarthritis on D20 and was euthanised D21 using a captive bolt gun and subsequent exsanguination.

### Faecal oocyst count (FOC)

**Fig. 2:**
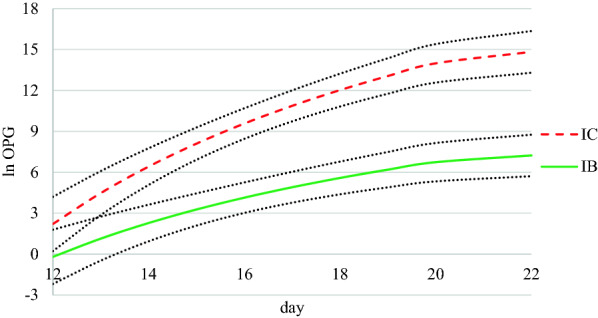
Estimated values of *Eimeria* oocysts per gram faeces (OPG) over time in lambs infected with *Eimeria* spp. (D0–D2), treated with spruce bark extract (IB) or treated with water (IC). The bark extract was given D0–D11. Area between dashed lines: 95% confidence interval. The figure shows data transformed with the natural logarithm (ln)

The lambs in IC started excreting oocysts on D14, and those in IB on D15, with 88% and 63% of the lambs excreting oocysts in group IC and IB, respectively. There was an extract by day interaction where the difference in oocysts per gram (OPG) between IB and IC increased with time (*P* < 0.05, Fig. 2). Compared to IC, IB had a lower mean oocyst count on D14 and onwards (*P* < 0.001), with an arithmetic mean oocyst count on D22 of 25,838 OPG in IB and 613,250 OPG in IC (SEM 16,854 and 173,190, respectively).

### Faecal consistency score (FCS)

**Fig. 3:**
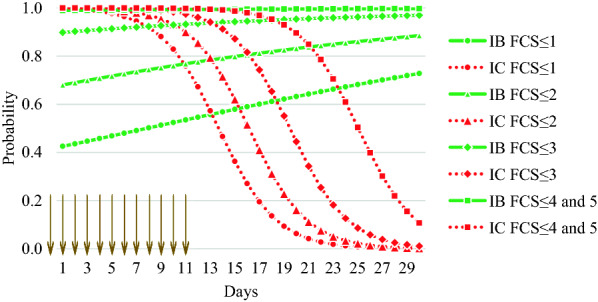
Estimated probability for faecal consistency scores (FCS) being less than or equal to 1, 2, 3, or 4 and 5 in lambs infected with *Eimeria* spp., treated (IB) or not treated with (IC) bark extract. The vertical arrows symbolise the time when bark extract was administered (D0–D11). IB and IC were infected for three consecutive days (D0–D2). FCS 1: hard pellets, 2: soft, sticky pellets, 3: soft, paste-like faeces with no pellet structure, 4: watery faeces, 5: watery faeces with blood and/or intestinal tissue

Before the start of the experiment (D0), all animals had pelleted, dry faeces. During the trial period (D0–D22), FCS differed between the two infected groups. From D0 to D11, IB lambs had higher FCS compared to IC, with 4 lambs (13%) in IB having FCS > 3 (i.e. diarrhoea) on D11 vs*.* 0 lambs in IC. For the post treatment period (D12-D22), the number of incidents with FCS > 3 was 1 vs*.* 22 in the IB lambs compared to IC lambs (Additional file 1). Thus, during the post treatment period, the animals treated with bark extracts (IB) were less prone to diarrhoea compared to the untreated animals (IC). This is illustrated in Fig. 3, which shows the estimated probability for FCS being less than or equal to a certain value. For IB, the probability of a decrease in FCS (i.e. more solid faeces) was lower at the beginning of the experiment compared to after D11, and the probability of a lower FCS increased with time. For lambs in IC, on the other hand, we observed a high probability of low FCS during the prepatent period (before D11), but this probability decreased with time (i.e. the animals were more likely to experience diarrhoea as time passed). The estimated parameters of treatment effect ($\alpha_{IB}$) and effect of treatment-day interaction ($\gamma_{IB}$) for IB were − 6.2 and 0.5, respectively, and day effect (*β*) for both IB and IC was -0.4. The equivalent parameters for IC, $\alpha_{IC}$ and $\gamma_{IC}$, were 0. Our null hypothesis ($H_{0} :\alpha_{1} = \alpha_{2} {\text{ and }}\gamma_{1} = \gamma_{2}$) that treatment with bark extract had no effect on FCS and that there was no extract-day interaction, had to be rejected (*P* < 0.001).

### Weight gains and milk consumption

There was a significant day by extract interaction on mean BW (*P* < 0.05). At D0, there was no difference in BW between the groups (*P* > 0.05). On D21, IB had a lower mean BW compared to IC and SC (*P* < 0.05). From D0 to D22, the estimated mean daily weight gain was 292 g/D, 387 g/D, and 415 g/D for IB, IC, and SC, respectively, with IB lambs having a lower weight gain than SC (*P* < 0.05).

There was a significant day by extract interaction on milk consumption (*P* < 0.05). IB had a lower milk consumption compared to IC during the whole treatment period (D0–D11) (Fig. 1). Compared to SC, IC experienced a reduction in milk consumption during D0–D2 when they were inoculated with *Eimeria* oocysts (Fig. 1), although this difference was not significant (*P* > 0.05). Additionally, this group had a minor drop in milk consumption from D13 to D19, also not significant (*P* > 0.05). SC consumed the milk they were offered during the whole experimental period.

## Discussion

This study showed that bark extract from Norway spruce had anticoccidial properties in milk-fed lambs infected with *Eimeria* spp. The regression analyses support the hypothesis that oral administration of bark extract reduces the excretion of *Eimeria* oocysts in lambs for the period up until 22 days after infection, corresponding to 12 days after the last day of bark extract administration. We also demonstrated that the difference in oocyst excretion between the treated and the untreated group increased with time, past the time of the extract administration. These findings agree with the results of other studies addressing the possible effects of CT containing PSM against protozoa. In a study where experimentally infected weaned lambs were fed CT-rich sainfoin (*Onobrychis viciifolia*), they found reduced faecal oocyst excretion in treated lambs compared to untreated control lambs [24]. Similarly, in an experiment with naturally infected goats it was found that animals receiving pine needles had significantly lower FOC than the untreated control group [29]. Quebracho extract supplemented to the diet reduced the *Eimeria* oocyst excretion in naturally infected goats [28]. Providing DM digestibility of a diet does not change, reduced feed intake will result in a reduced amount of faeces. Therefore, FOC might appear to increase as it is a concentration number. Similarly, diarrhoea will have a diluting effect on the oocyst concentration, as FOC is estimated on fresh matter basis. To reduce this type of biases, it would have been ideal to calculate faeces dry matter content and express the oocyst number per gram of dry matter faeces. However, it is important to point out that both a lower feed intake and lower FCS in group IB would increase FOC and not decrease it, as we observed.

After discontinuation of the treatment (from D12 onwards), the lower FCS of IB coincided with the lower FOC, compared to IC. From D14, we saw that IC had a higher FCS compared to IB and SC, which coincided with the prepatent time of ovine *Eimeria* infections, which is 12–20 days for most ovine species, including the pathogenic species *E. ovinoidalis* and *E. crandallis* that constituted ≈ 62% of the infection dose used in our study [32]. A high oocyst excretion correlates with damage to the intestinal mucosa and diarrhoea in ruminants [38].

The IC lambs had a reduction in milk consumption during the oocyst inoculation period (D0–D2), which can be expected for animals exposed to parasites [39]. Additionally, this group had a drop in milk intake during D13–D19. This period coincides with the end of the prepatent period of several ovine *Eimeria* spp., which is when we would expect damage to the intestinal mucosa and a subsequent reduction in appetite [38]. IB lambs also had a reduction in milk intake in the same time frame, but to a much lesser degree and on D13–D14 only.

Shortly after bark extract treatment, several of the animals in the IB group had reduced milk intake and showed symptoms of obstipation and colic, in some cases with subsequent diarrhoea. We also observed lower mean daily weight gain for IB compared to IC and SC lambs. We assumed that the elevated FCS and the reduction in milk consumption during the treatment period (D0–D11) was a consequence of the bark extract administration, which was confirmed by the fact that FCS decreased, and the appetite returned to normal after discontinuation of the extract treatment. The reduced growth we experienced in this trial was probably due to the reduction in voluntary feed intake, likely attributed to the high astringency of CTs, hence the low palatability of the extract [40]. Additionally, direct toxic effects of the extract, or impact on the dry matter digestibility of the feed, can have a negative impact on the animal’s growth [41, 42].

Detrimental effects of plant extracts have been reported previously. For instance, CT-rich quebracho extracts have shown to cause anorexia, diarrhoea, and reduced growth in lambs [43, 44]. The observed difference in BW between the treatment groups in these two studies was nullified at the end of the experiment, day 39–67. In the present trial, the animals were euthanised at D22, hence we were unable to evaluate the long-term effect of the bark extract on the BW.

In another experiment, extracts of Ethiopian plants caused anorexia and near moribund behaviour in house mice infected with *Heligmosomoides bakeri* [45]. Loose stool in sheep and goats treated with wattle tannin extracts containing 70% CT was attributed to the low molecular weight of hydrolysable tannins in the extract, the ability of hydrolysable tannins to desquamate the intestinal surface epithelium and in this way cause diarrhoea [46]. Contradictory to these findings, in trials where lambs were naturally infected with *Eimeria* spp. were fed CT-rich sainfoin diets, there were no differences between the groups regarding FCS and weight gain [24, 27]. Although the effective CT dose in our trial (2.4–4.8 g CT/20 kg BW) was lower than that used in other studies (10–58 g CT/20 kg BW) [24, 47, 47], our IB lambs still exhibited detrimental effects of the bark extract, i.e. lower body weight gain, reduced milk consumption, and increased FCS during the treatment, compared to IC. Possible reasons for this might be the different composition of the plant materials (spruce bark *vs.* sainfoin vs*.* wattle vs. quebracho), the route of administration of the CT-rich supplement (extract vs*.* in-feed), or the age of the animals (pre-weaned *vs.* weaned). There might be other, non-CT, components in the respective plants contributing to the antiparasitic effect.

A reduction in the levels of parasitism is often followed by improvement on the performance of the host. In this trial, although we saw a significant reduction in OPG and a lower FCS of IB lambs in the post treatment period, we also observed detrimental effects of the extracts, such as higher FCS during the treatment period and a significant reduction in milk consumption and weight gain. It has been previously reported that CT consumption can have positive (antiparasitic) and negative (anti-nutritional) consequences when consumed by parasitised host [48]. Although IB had significantly lower overall FOC (D1–D22) compared to IC (98.0% lower), the anti-nutritional effects of the bark extracts on performance (10.3% lower BW for IB at the end of experiment and 29.0% lower milk intake for IB for the treatment period (D0–D11), compared to IC) and the initial problems of indigestion outweighed the pathological effects of the coccidia infection, thus the trade-off resulted in an overall cost for the parasitised animals. A reduction in the extract dose, in volume and/or in CT content per kg BW, or in the duration of administration, would most likely have resulted in a better trade-off between anti-parasitic efficacy and adverse effect induced by the supplementation in the treated lambs. Nevertheless, the possibility that the BW of the treated lambs may have caught up with or surpassed that of lambs in the sham-infected control group, if the experiment was prolonged, cannot be excluded.

CT are components important for the plant’s protection; their consumption can penalise herbivores and act as feeding repellents. The consumption of tannins has been associated with a reduction in feed intake and dry matter digestibility when administered at 7.5–10% of the dry matter [42]. High concentrations of Quebracho extract are considered toxic to ruminants which can be attributed to CTs’ ability to bind to proteins [49]. It seems that CT administered via the feed, e.g., as browse, hay, or pelleted feed, may have fewer negative effects on faecal consistency and feed intake compared to crude plant extracts administered through drenching [24, 27, 43, 45, 46, 50]. The animals in our trial were young lambs without a fully developed ruminating function. It has been reported that ruminants are able to tolerate potential toxic effects of CT by slowly adapting their ruminal bacteria through shifting the microbial population towards microbes able to alter the CT [51]. It is likely that young lambs with undeveloped rumen may not have this ability, which would make them more susceptible to an astringent, toxic component. Another mechanism for tolerating the anti-nutritional effects of CT is by excreting CT-binding proteins in the saliva [40]. Little research has focused on CT-binding proteins in ruminant saliva, and it is plausible that the protein composition in pre-ruminant lambs is different from adults.

It is improbable that the reduction in milk intake in IB lambs was due to the total volume of extract offered. According to Large, 1964, the volume of omasum-abomasum measured in ≥ 3 weeks old lambs of a similar breed is > 250 mL, which was the amount of bark extract given in our trial [52, 53]. This was indeed confirmed by the fact that IC receiving 250 mL water consumed all the milk replacement they were offered. Nevertheless, the bark extract had a higher viscosity compared to water, which might have influenced the passing time of the drenched substance (i.e. the time it takes for the extract to pass the abomasum). Furthermore, the bark extract contains components (e.g., CT) which might influence the abomasum and the animal negatively, e.g., by causing nausea.

In this trial, the bark extract was administered by oral gavage for 12 days. More research is needed regarding modes of administration to make it practicable to use bark extract as an agent to control parasites in young lambs.

We suggest that future research should focus to improve the trade-off between the negative side effects *vs.* the benefits of the treatment to assess if the trade-off could be better utilised at a lower bark extract concentration. We also recommend conducting a dose–response study and to evaluate the outcome with subsequent necropsy. Furthermore, we suggest additional studies with higher parasite load and a longer follow-up period, e.g., from birth till slaughtering at 5 months of age, which is common in Norway, to assess if the bark extract treated group will benefit from the lower parasite burden in a longer perspective.

Coccidiosis is considered an important disease in sheep production worldwide. If spruce bark extracts can be used to control coccidiosis in sheep, it will help reducing oocyst shedding and prevent the contamination of pastures. This will decrease the need of metaphylactic treatment, which again will reduce the development of anticoccidial resistance.

## Conclusion

The water-based extract of bark from Norway spruce (*P. abies*) showed anticoccidial properties by reducing the oocyst excretion in milk-fed lambs. Additionally, after treatment with bark extract, the lambs experienced a lower incidence of diarrhoea. We observed unwanted side effects of the bark extract, expressed as cases of indigestion, reduced milk consumption and body weight gain.

## Supplementary Information

**Additional file 1** Faecal consistency score of every individual on each sampling day. IB and IC were infected for 3 consecutive days (D0–D2), with 100,000 sporulated *Eimeria* oocysts. IB was treated with spruce bark extract for 12 consecutive days (D0–D11). IC and SC were given the equivalent amount of tap water. SC was not sampled on D10–D19. FCS: faecal consistency score; IB: infected lambs treated with bark extract; IC: infected, untreated control animals; SC: sham-infected, untreated control animals. n is the number of samples collected on the specific sampling day.

## Data Availability

The datasets used and analysed during the current study are available from the corresponding author on reasonable request.

## Abbreviations

BW: Body weight
CT: Condensed tannins
DM: Dry matter
FCS: Faecal consistency score
FOC: Faecal oocyst count
IB: Infected lambs treated with bark extract
IC: Infected, untreated control animals
SC: Sham-infected, untreated control animals
mBW: metabolic body weight
OPG: Oocysts per gram faeces
PSM: Plant secondary metabolite
